# Nitric oxide increases biofilm formation in *Saccharomyces cerevisiae* by activating the transcriptional factor Mac1p and thereby regulating the transmembrane protein Ctr1

**DOI:** 10.1186/s13068-019-1359-1

**Published:** 2019-02-14

**Authors:** Leyun Yang, Cheng Zheng, Yong Chen, Xinchi Shi, Zhuojun Ying, Hanjie Ying

**Affiliations:** 1 National Engineering Research Center for Biotechnology, College of Biotechnology and Pharmaceutical Engineering, Nanjing Tech University, Nanjing, China; 2 State Key Laboratory of Materials-Oriented Chemical Engineering, College of Biotechnology and Pharmaceutical Engineering, Nanjing Tech University, Nanjing, China; 3 College of Life Science, Nantong University, Nantong, China; 4 University of California, San Diego, USA

**Keywords:** *Saccharomyces cerevisiae*, Nitric oxide, *MAC1*, *CTR1*, Biofilm

## Abstract

**Background:**

Biofilms with immobilized cells encased in extracellular polymeric substance are beneficial for industrial fermentation. Their formation is regulated by various factors, including nitric oxide (NO), which is recognized as a quorum-sensing and signal molecule. The mechanisms by which NO regulates bacterial biofilms have been studied extensively and deeply, but were rarely studied in fungi. In this study, we observed the effects of low concentrations of NO on biofilm formation in *Saccharomyces cerevisiae.* Transcriptional and proteomic analyses were applied to study the mechanism of this regulation.

**Results:**

Adding low concentrations of NO donors (SNP and NOC-18) enhanced biofilm formation of *S. cerevisiae* in immobilized carriers and plastics. Transcriptional and proteomic analyses revealed that expression levels of genes regulated by the transcription factor Mac1p was upregulated in biofilm cells under NO treatment. *MAC1* promoted yeast biofilm formation which was independent of flocculation gene *FLO11*. Increased copper and iron contents, both of which were controlled by Mac1p in the NO-treated and *MAC1*-overexpressing cells, were not responsible for the increased biofilm formation. *CTR1*, one out of six genes regulated by *MAC1*, plays an important role in biofilm formation. Moreover, *MAC1* and *CTR1* contributed to the cells’ resistance to ethanol by enhanced biofilm formation.

**Conclusions:**

These findings suggest that a mechanism for NO-mediated biofilm formation, which involves the regulation of *CTR1* expression levels by activating its transcription factor Mac1p, leads to enhanced biofilm formation. The role of *CTR1* protein in yeast biofilm formation may be due to the hydrophobic residues in its N-terminal extracellular domain, and further research is needed. This work offers a possible explanation for yeast biofilm formation regulated by NO and provides approaches controlling biofilm formation in industrial immobilized fermentation by manipulating expression of genes involved in biofilm formation.

**Electronic supplementary material:**

The online version of this article (10.1186/s13068-019-1359-1) contains supplementary material, which is available to authorized users.

## Background

Biofilms are communities of microbes embedded within self-produced extracellular polymeric substances [1]. Members of a biofilm community have better survival under stress caused by adverse environmental conditions, and can cause considerable damage in many industrial and clinical settings [2]. For example, biofilm growth in drinking water systems can result in pipe corrosion, generation of bad tastes and odors, and proliferation of pathogens [3]. In the clinical context, biofilms formed by pathogens on host tissues and artificial surfaces can result in troublesome persistent infections [4]. However, the positive characteristics of biofilms have also been exploited in many fields. In the sewage-treatment industry, biofilms are grown on carriers to remove heavy metals [5]. Immobilization technology to form biofilms of industrial stains like *Saccharomyces cerevisiae*, *Sporolactobacillus inulinus,* and *Clostridium acetobutylicum* on carriers has been applied to efficiently produce biochemical products [6–8]. In fuel-ethanol production, the repeated batch fermentation of *S. cerevisiae* in a biofilm reactor reached a higher conversion rate than free fermentation. In addition to a high yield of ethanol, a short fermentation cycle and excellent tolerance to ethanol were observed during this fermentation process [6].

The switch from planktonic lifestyle to biofilm formation goes through three phases—attachment, maturation, and dispersion. Statistical analysis revealed that biofilm genes show significant expression changes mainly during attachment, which underscores the importance of the attachment period in biofilm formation [9]. Biofilm formation is influenced by various extrinsic and intrinsic factors. Glucose, sodium chloride, pH, temperature, and nutrients are common environmental factors affecting biofilm formation [10]. As intrinsic factors, quorum-sensing (QS) molecules have recently gained attention for their role in biofilm formation. QS is defined as a cell–cell communication process that involves the production, detection, and response to small extracellular signaling molecules called autoinducers (AIs) [11]. Various processes including motility, virulence, competence, conjugation, and sporulation, especially biofilm formation, are controlled by QS [12, 13]. Biofilms are structured communities of cells that are regulated by QS via controlled communication of the constituent cells. *N*-acylhomoserine lactones are major QS molecules in Gram-negative bacteria, and play an important role in biofilm formation [14]. In yeast, several aromatic alcohols such as tryptophol, phenylethyl alcohol, and farnesol were shown to act as AIs in QS [15, 16]. For example, farnesol, a QS molecule found in fungi, inhibits biofilm formation of *Candida albicans* [17].

Gaseous nitric oxide (NO) was also identified as a QS molecule in many studies [18, 19]. NO is a free radical that can freely diffuse in biological system [19]. Exposure to low concentrations of exogenous NO has been shown to result in different responses in biofilm formation. In several bacterial species, such as *Pseudomonas aeruginosa*, *Staphylococcus epidermidis*, and *Escherichia coli*, NO triggers biofilms’ dispersion [20–22]. By contrast, NO was found to enhance biofilm formation of *Vibrio harveyi* and *Shewanella oneidensis* [18, 23]. Interestingly, the exogenous addition of NO with NO donors had no effects on biofilm formation in *Bacillus subtilis* [24]. The regulatory pathway that mediates the effects of NO in bacterial biofilm formation is well studied. In bacteria, NO is detected by a selective NO sensor—the heme-nitric oxide/oxygen-binding domain of soluble guanylate cyclase (sGC)—which goes on to modulate biofilm formation by controlling the levels of the second messenger cyclic di-GMP (c-di-GMP) [23, 25].

In fungi, various NO donors have been used to study the role of NO in biofilm formation, but the mechanism of this regulation has not been described in detail. For example, the adhesion and biofilm formation of *C. albicans* cells was observed to be inhibited by NO [26]. The intracellular concentration of the second messenger cyclic GMP (cGMP) has been found to be regulated by NO in *Coniothyrium minitans* [27]. It is highly likely that cGMP in fungi plays a role akin to that of c-di-GMP in bacteria, mediating the regulation of biofilm formation by NO. However, cGMP has been detected in only a few fungi such as *Phycomyces blakesleeanus*, *Neurospora crassa*, and *Blastocladiella emersonii* [28]. In fact, it is also unclear whether cGMP can be synthesized in yeast, because the yeast genomes, including those of *Schizosaccharomyces pombe*, *Candida albicans*, and *S. cerevisiae,* lack homologs to guanylate cyclase genes, and no studies have reported the presence of cGMP in yeast since 1980s [29]. Moreover, the approaches used to investigate the NO-mediated regulation of biofilm formation in bacteria are not appropriate for yeasts.

We report for the first time that *S. cerevisiae* biofilm formation was increased by the addition NO donors. To understand the mechanisms mediating the influence of NO on biofilm formation, comparative transcriptomic and proteomic analyses of NO-treated biofilm cells and untreated control were performed. We found that genes/proteins downstream of the transcription factor Mac1p were all upregulated at both the RNA- and protein-expression levels. *MAC1* contributed to yeast biofilm formation, whereby this process did not rely on changing the expression level of *FLO11* and was not related to the changed copper and iron contents in the cells. Among six downstream genes regulated by *MAC1*, only *CTR1* contributed to yeast biofilm formation. In addition, biofilm cells of mutants overexpressing *MAC1* and *CTR1* showed increased ethanol resistance and fermentation rates, especially in the later stages of fed-batch fermentation. Thus, it can be concluded that NO regulates yeast biofilm formation by activating the transcriptional factor Mac1p and thereby controlling the expression level of *CTR1*.

## Materials and methods

### Yeast strains and growth conditions

*Saccharomyces cerevisiae* 1308 [30] is a diploid industrial strain isolated from fermentative habitats and maintained on conventional YPD agar plates. The fermentation medium was optimized and contained glucose (200 g/L), peptone (4 g/L), (NH_4_)_2_SO_4_ (4 g/L), yeast extract (3 g/L), KH_2_PO_4_ (3 g/L), MgSO_4_ (0.5 g/L), ZnSO_4_·7H_2_O (0.05 g/L), and FeSO_4_·7H_2_O (0.05 g/L). To select yeast transformants, G418 Sulfate (345180; Merck, Japan) was added to make final concentrations of 400 and 800 µg/mL to solid yeast extract peptone dextrose medium (YPD).

Seed cultures were grown at 30 °C in 250-mL Erlenmeyer flasks containing 30 mL YPD medium in a rotary shaker at 200 rpm. Fermentations were performed by adding 1 mL overnight cultures into 250-mL flasks containing 100 mL of fermentation medium with or without the addition of 4 g of dry cotton fiber for biofilm attachment. Sodium nitroprusside dihydrate (SNP; Sangon Biotech, China) was added to the flasks to make final concentrations of 0–300 μM. Flasks were placed on a shaker at 250 rpm and maintained at 35 °C. Continuous batch fermentation was conducted for the immobilized culture, whereby “waste broth” was removed and fresh broth was added as residual glucose was depleted (< 1 g/L). Samples were drawn from each flask at 4-h intervals. Cell concentrations were determined spectrophotometrically by measuring the OD_600_. The glucose concentration of the supernatant was tested using the DNS (3,5-dinitrosalicylic acid) method. The ethanol concentration was analyzed by gas chromatography using an Agilent HP-INNOWAX column (60 m × 250 μm × 0.5 μm) as described previously [30].

### Transcriptomic analysis

Biofilm cells were isolated from cotton fibers via ultrasonication during the biofilm attachment period (3 h) and then washed twice in PBS. Cell pellets were immediately frozen in liquid nitrogen and stored at − 80 °C. Three biological replicates were prepared from the samples taken under NO-treated and untreated conditions. RNA was isolated from biofilm *S. cerevisiae* cells using the methods described previously, and a cDNA library was constructed using published methods [9]. The reads per kilobase transcriptome per million mapped reads method (RPKM) was applied to calculate the expression levels of selected genes. This study selected a level of FDR ≤ 0.001 and absolute value of Log_2_Ratio ≥1 as criteria for assessing the significance of differential gene expression. The Illumina sequencing data were deposited into the NCBI database under the accession number SRP153792.

### Proteomic analysis

Biofilm cells were harvested and prepared in the same way as for transcriptomic analysis. Protein extraction, iTRAQ labeling (isobaric tag for relative and absolute quantitation, mass spectrometry, and database searching were performed as described previously [31]. Cells were lysed using the glass bead-shaking method in lysis buffer comprising 100 mM DTT, 5% SDS, and 0.1 M Tris–HCl (pH 7.6). The lysate was centrifuged at 13,000×*g* for 10 min, and the supernatants were collected. The extracted proteins were quantified using the 2-D Quant Kit (GE Healthcare, USA). Trypsin digestion was performed using trypsin (Promega, USA) according to the manufacturer’s instructions. The peptides were ionized by an NSI source followed by tandem mass spectrometry (MS/MS) in Q ExactiveTMPlus (Thermo Fisher Scientific, USA) coupled online to a UPLC (Shimadzu, Japan). ProteinPilot™ Software 4.5 (AB SCIEX) equipped with Paragon Algorithm was used for data processing. The software performed automatic recalibration such that typical mass errors for MS and MS/MS data were below 5 ppm and the mass tolerance for fragment ions was set as 0.02 Da. Comparative protein data with ratios of > 1.2 and < 0.833 with *p*-values < 0.001 were identified as showing differential expression. The mass spectrometry proteomics data have been deposited to the ProteomeXchange Consortium with the dataset identifier PXD010751.

### Construction of overexpression and deletion mutants

*Saccharomyces cerevisiae* knockout mutants were constructed by deleting corresponding genes in *S. cerevisiae* 1308, the selected industrial yeast strain, using the homologous recombination system (LFH-PCR: PCR synthesis of disruption cassettes with long flanking homology) according to published methodology [30]. The PCR-generated DNA molecules (knock-in components) consisted of a KanMX marker cassette, for G418 resistance in *S. cerevisiae* and kanamycin resistance in *Escherichia coli*. KanMX marker cassettes with long homologous arms (450–500 bp) flanking the target locus were then used for directed gene alterations in *S. cerevisiae*. The amplified knock-in components were then electroporated into competent *S. cerevisiae* 1308 cells produced using the sorbitol method using a electroporation system (Bio-Rad, USA) set at 1.5 kV, 25 mF with a 200 Ohm pulse controller. Genes were amplified and inserted into pYX212 using the ClonExpress One-Step Cloning Kit, respectively (Vazyme Biotech, Nanjing, China). The plasmids were transformed into the WT strain, using G418 (400 μg/mL) to select stably transfected clones. The PCR primers used in this study are listed in Table 1.

**Table 1:** Sequences of the oligonucleotide primers used in this study

Primer name	Primer sequence	Source
MAC1-up-F	AATGGGAACAAATATGCGTGTGCATCGTGCATCAG	This work
MAC1-up-R	GCCTCCATGTCCGTATAGGCTCCTGTTGAAGCC	This work
MAC1-dn-F	GCTGGTCGCTATACTGTCCTTGGAATCTACGTC	This work
MAC1-dn-R	ATCCGGAGGACATATGCATTCCTTGTCAGTGCATTTAC	This work
G418-MAC1-F	GGAGCCTATACGGACATGGAGGCCCAGAATAC	This work
G418-MAC1-R	CGTAGATTCCAAGGACAGTATAGCGACCAGCATTCAC	This work
CTR1-up-F	GATGTCTAGTGCCAGCAAAACGATATTATCG	This work
CTR1-up-R	CCTCCATTGTCTGGAGTTTGCTGAAGGTAAA	This work
CTR1-dn-F	CTGGTCGCTATACTGGACACAGAGAATAATT	This work
CTR1-dn-R	GTTATGAGTGAATTTTTCGGCCGGAAG	This work
G418-CTR1-F	AAACTCCAGACATGGAGGCCCAG	This work
G418-CTR1-R	AATTATTCTCTGTGTCCAGTATAGCGACCAGC	This work
CTR3-up-F	TATGGGAGGCAGTAGCAGCACTGCT	This work
CTR3-up-R	CTGGGCCTCCATGTCAAAGCCTTGTAGTTC	This work
CTR3-dn-F	CGCTATACTGTACAACGAACCAAGCTGGAA	This work
CTR3-dn-R	ACAAGCAGCATTTGCGATCATCACTCTCT	This work
G418-CTR3-F	TACAAGGCTTTGACATGGAGGCCCAGAAT	This work
G418-CTR3-R	CAGCTTGGTTCGTTGTACAGTATAGCGACC	This work
FRE1-up-F	CGGTTCAATCGAGTGCTACACTTATTAGC	This work
FRE1-up-R	CTGGGCCTCCATGTCTCAAGATAGTGGCTGCAG	This work
FRE1-dn-F	GGTCGCTATACTGTGGTAAGAACATCATGG	This work
FRE1-dn-R	AACGGCCAACATGAAACAAACGTAGGC	This work
G418-FRE1-F	CCACTATCTTGAGACATGGAGGCCCAGAA	This work
G418-FRE1-R	TGATGTTCTTACCACAGTATAGCGACCAGC	This work
FRE7-up-F	TATTGCTGACATCCACTCCGAACTATACGC	This work
FRE7-up-R	GGCCTCCATGTCGGTCTGTAGAATGGA	This work
FRE7-dn-F	TCGCTATACTGCGCCTTTGTCTGTTCG	This work
FRE7-dn-R	ACCAAACCACAATTGTAGCAACCAGATAC	This work
G418-FRE7-F	ATTCTACAGACCGACATGGAGGCCCAGAATA	This work
G418-FRE7-R	CAGACAAAGGCGCAGTATAGCGACCAG	This work
IRC7-up-F	CGCAACTGTCTGTTATTGGACGTAATCCAG	This work
IRC7-up-R	GCCTCCATGTCCCTATTGATGGGTCATAA	This work
IRC7-dn-F	CTGGTCGCTATACTGGTTAAGCCAAATACAAC	This work
IRC7-dn-R	AACCCAGTATTCATGTCCGGGACAATCTTC	This work
G418-IRC7-F	ACCCATCAATAGGGACATGGAGGCC	This work
G418-IRC7-R	TTGTATTTGGCTTAACCAGTATAGCGACCAG	This work
REE1-up-F	GAATACTGAATTATCGCAAGGAACATGGCT	This work
REE1-up-R	CTGGGCCTCCATGTCCCATCAGAAATTTC	This work
REE1-dn-F	GGTCGCTATACTGTTTTGACAAATGGAAAATCAG	This work
REE1-dn-R	CAAATCATGTAAAGCTTTTCCTAAAGGAGCTG	This work
G418-REE1-F	AATTTCTGATGGGACATGGAGGCCCAG	This work
G418-REE1-R	TTCCATTTGTCAAAACAGTATAGCGACC	This work
pAurR-*MAC1*-F	TTCAGTTAGCTAGCATGATAATATTTAATG	This work
pAurR-*MAC1*-R	GTGCCACCTGACGTCTTATGAAGTGGTGGCA	This work
pAurR-*CTR1*-F	TTCAGTTAGCTAGCATGGAAGGTATGAATAT	This work
pAurR-*CTR1*-R	GTGCCACCTGACGTCTTAGTTATGAGTGAA	This work
pAurR-*CTR3*-F	TTCAGTTAGCTAGCATGAATATGGGAGGCAGT	This work
pAurR-*CTR3*-R	GTGCCACCTGACGTCTTAGTTATGAGTGAA	This work
pAurR-*FRE1*-F	TTCAGTTAGCTAGCATGGTTAGAACCCGTGT	This work
pAurR-*FRE1*-R	GTGCCACCTGACGTCTTACCATGTAAAACTTTC	This work
pAurR-*FRE7*-F	TTCAGTTAGCTAGCATGATTGAAGAAAGAG	This work
pAurR-*FRE7*-R	GTGCCACCTGACGTCCTAGTAGCCAAAACTCTCG	This work
pAurR-*IRC7*-F	TTCAGTTAGCTAGCATGATTGATCGTACCGAGTTA	This work
pAurR-*IRC7*-R	GTGCCACCTGACGTCCTAGCCACCCCATGAAATCCC	This work
pAurR-*REE1*-F	TTCAGTTAGCTAGCATGGTCGAATCTAAGAA	This work
pAurR-*REE1*-R	GTGCCACCTGACGTCCTAACTCAAATCATGTAAAG	This work

### qRT-PCR analysis

Reverse transcription was performed using an AMV First Strand cDNA Synthesis Kit (Sangon Biotech, China) according to standard protocols. Primer 5 software was used to select the primers. The analyzed genes and primers used for analysis are listed in Table 2. Quantitative real-time PCR (qRT-PCR) assays were performed using SYBR Green PCR Master Mix (Applied Biosystems, USA) in a StepOnePlus Real-Time PCR System (Applied Biosystems, USA). Reactions were performed according to the manufacturer’s instructions, and three technical replicates with one negative control were performed for each sample. Gene transcription levels were determined according to the 2^−∆∆CT^ method, using the 18S rRNA as reference gene for normalizing gene expression levels [9].

**Table 2:** Genes and primers used for quantitative real-time PCR

Gene	Forward primer sequence (5′–3′)	Reverse primer sequence (5′–3′)
*MAC1*	TGCTGCAGCGCAATGAA	TCTAACAGCAGAGGCACGTACAA
*CTR1*	CGGTAACTGCCAATGTGGTAGA	ATCGGCAACAGCAATTGGAT
*CTR3*	CGGCTGTTTTGCGCTTGT	TCAAATTGCCTTGAAAAACGAGTA
*FRE1*	AATGGTCTGCCTACGTTTGTTTC	CGAGGCGGTCATGACAATT
*FRE7*	TGGCCTCGACCATTGCA	GACGATCAATTCTACGCATCCTT
*IRC7*	GGCTCGGAAATCGAGATGAG	TCCGGGACAATCTTCAAAGG
*REE1*	TCCATTTCCAACTTCTGACCATT	CCACGCTCAGGTGTGCAA
*FLO11*	ACTTTGGATGTGACTTCCGTTTC	ACCTTTGACATGAATAGTGATTTGGTA
*18S*	ACGGAGCCAGCGAGTCTAAC	CGACGGAGTTTCACAAGATTACC

### Biofilm formation on plastics

Yeast strains were grown in YPD overnight at 30 °C. After collection and washing, cells were resuspended in YPD at an OD_600_ of 1 and transferred to the wells of a 96-well microtiter plate (Corning, NY) where they were incubated for 24 h at 30 °C. SNP, diethylenetriamine NONOate (NOC-18, ≥ 98%; Cayman, USA), NaNO_2_ and NaNO_3_ were individually added to wells to final concentrations from 0 to 300 μM. Carboxy-PTIO (cPTIO, > 98.0%; TCI, Japan) potassium salt was added to another group containing SNP, at a final concentration of 1 mM. Four replicate wells were used for each treatment. Biofilm-containing wells were washed twice with 200 μL PBS to remove free cells, after which the biofilms were stained with 1% crystal violet, followed by repeated washing of the wells with water and photographic recording. For quantitation, crystal violet was solubilized by adding 100 μL of acetic acid, after which the plates were incubated for 15 min at room temperature, and the absorbance at 570 nm was measured using a microplate reader.

### Standard plate-wash assay

Cells were grown on standard YPD agar plates for 3 days. Observations indicated all strains grew equally well in this environment. Next, each plate was added to 1 mL water and shaken at 50 rpm for 2 min. The water was then discarded, and images of the colonies were recorded.

### Scanning electron microscopy (SEM) analysis

Biofilm cells were harvested after 24-h fermentation in the presence of SNP and control. Samples were washed twice with PBS buffer, and stored at − 80◦C. Biofilm cells were dried using a FreeZone^®^ 4.5 L Freeze Dry System (Labconco, KansasCity, MO, USA) and sputter coated with gold. Images were obtained using a Hitachi S-4800 field.

### Ethanol resistance test

First, yeast cells underwent a 1-day immobilization process on cotton fibers under the previously described immobilization conditions (see “Yeast strains and growth conditions” section). When the ethanol resistance test was started, 90 mL of fresh medium containing 45 g/L glucose and 10 mL of absolute ethanol were added (the other ingredients were the same as above), before the residual medium was discarded into flasks. Samples were drawn from each flask at 3-h intervals. The detection of glucose concentrations in samples was conducted as described above.

### Determination of the intracellular copper and iron contents

The copper and iron contents of cells were quantified using inductively coupled plasma mass spectrometry (ICP–MS) as described previously [32]. Biofilm cells were washed three times with phosphate-buffered saline (PBS) containing 0.5 mM EDTA. The cell pellets were dried overnight at 80  °C in 10-mL polytetrafluoroethylene tubes, and then subjected to acid digestion using 70% trace metal-grade nitric acid (Fluka, Sigma-Aldrich, USA) at 80 °C for 30 min, followed by cooling down to room temperature. The acid-digested samples were transferred to 50-mL Falcon tubes. Samples were diluted with Milli-Q water and mixed with the internal standards (^59^Co and ^89^Y) to yield final concentrations of 2 ppb internal standards and 1% nitric acid before the reaction mixture was subjected to quantitation via ICP-MS (Agilent 7500a; Agilent Technologies, USA).

### Statistical analysis

All experiments were done at least in triplicate. The data represent the means of three or more experiments. The significance of differences (*p* < 0.05) was determined using Student’s *t*-test in software version.

## Results

### Exogenous NO induced yeast biofilm formation

**Fig. 1:**
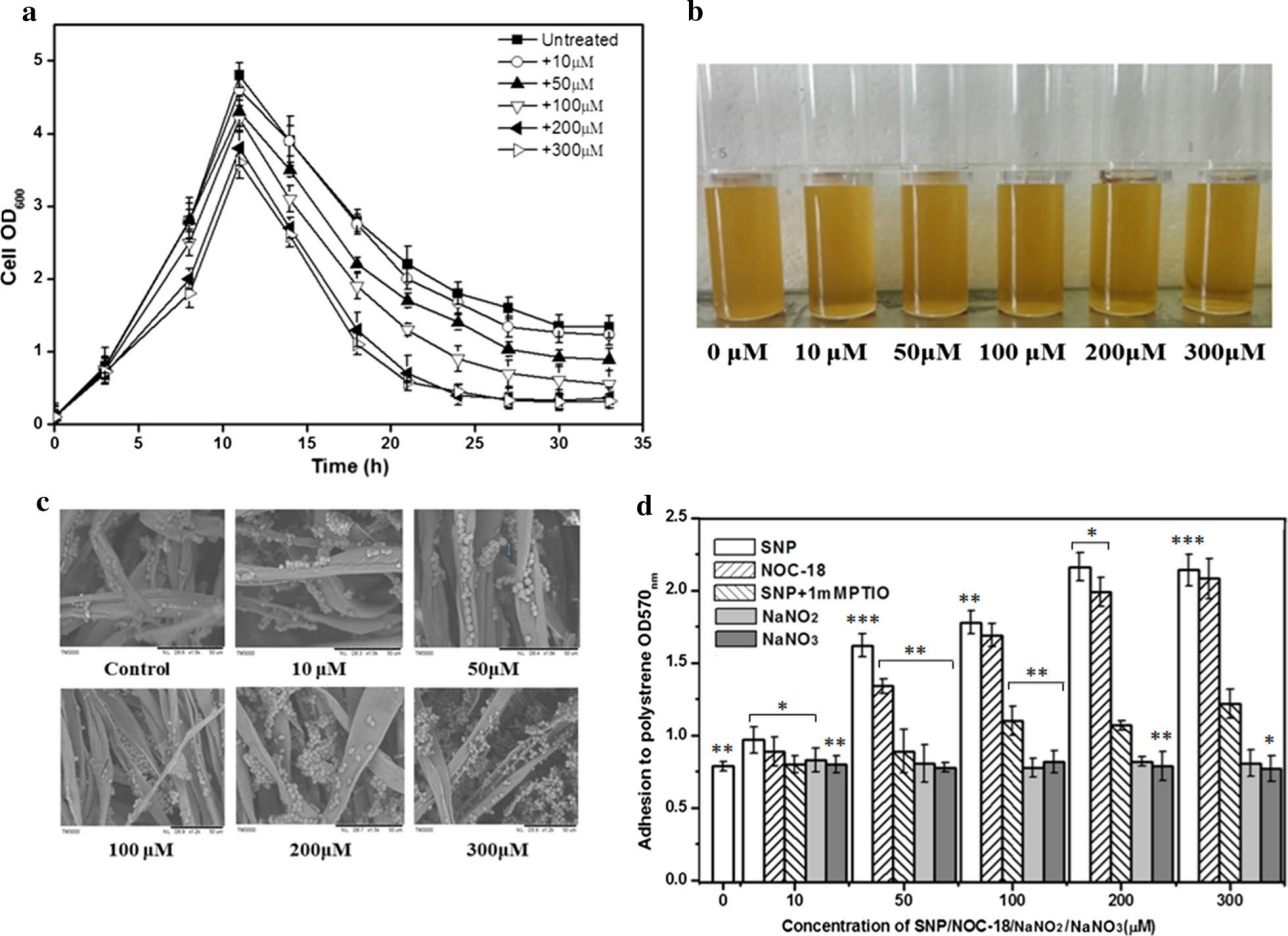
Biofilms formed under treatment with NO donors. **a** Growth curve of free cells in the presence of different concentrations of SNP during immobilized fermentation. **b** After 24 h of fermentation, the culture broths from the different groups were observed. **c** Biofilms formed on cotton fibers after fermentation imaged by SEM. **d** Biofilms formed in 96-well plates for 24 h in the presence of NO donors, SNP and NOC-18, and the scavengers PTIO, NaNO_2_, and NaNO_3_. The wells were washed twice with PBS (200 μL) to remove free cells and stained with 1% crystal violet. Biofilm formation was measured at 570 nm after solubilizing crystal violet in acetic acid. The values are the means and standard deviations of three independent experiments. ***p < 0.001, **p < 0.01, *p < 0.05 by Student’s *t*-test

A range of concentrations of the NO donor SNP (0–300 μM) which has no impact on cell growth (Additional file 1: Figure S1) were added to cultures with added cotton fibers. During the fermentation, the cell concentration in the liquid culture was decreasing in the presence of SNP, reaching a minimum at 200 μM SNP (Fig. 1a). At the end of fermentation, it could be observed that the fermentation liquids were increasingly clear and transparent with the increasing concentration of SNP (Fig. 1b). In addition, cell concentrations reached the minimum at 27 h in the presence of 200 and 300 μM SNP, compared to 30 h in the control. The biofilm formation under NO treatment entered into stabilization than control. To observe the biofilm formed on fibers under SNP stimulation, scanning electron microscopy was employed. Cells exposed to NO produced a remarkably thick biofilm on the fiber surface compared to the control (Fig. 1c). NO not only increased the amount of biofilm, but also raised the speed of its formation. These observations were in agreement with the quantitation of biofilm formation of *S. cerevisiae* grown in 96-well plates using the crystal violet staining method (Fig. 1d). Hence, more biofilm was formed in the 96-well plates with the SNP treatment than in the control. When the concentration was increased to 200 μM, biofilm formation was 2.7-fold higher than that of the untreated culture. Moreover, treatment with NOC-18, an alternative NO donor molecule that is chemically and mechanistically distinct from SNP, resulted in an increase of biofilm formation similar in extent to the treatment with SNP. Moreover, the enhancement of biofilm formation was abrogated in the presence of the NO scavenger cPTIO. The lack of response to SNP indicated that the biofilm strengthening effects were indeed mediated by NO instead of the degradation product prussiate. Treatment with nitrate or nitrite ranging from 50 to 300 μM failed to significantly enhanced yeast biofilm formation, indicating that the oxidative breakdown products of NO were not responsible for the effect of NO on biofilm formation.

### The transcription factor Mac1p was activated in biofilm cells treated with NO

**Fig. 2:**
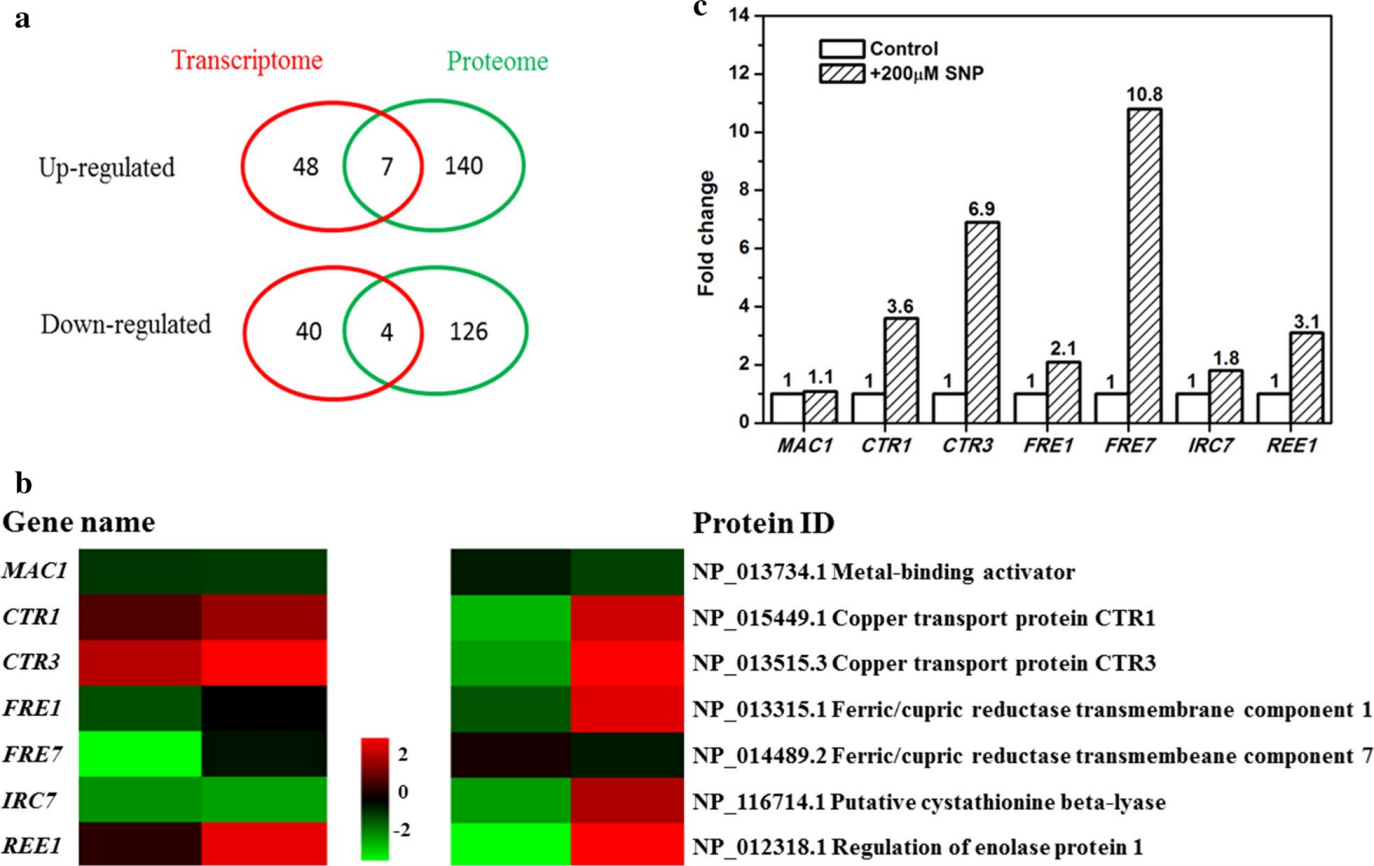
**a** Comparison of the transcriptomes and proteomes of cells that formed biofilms under control conditions and under treatment with 200 μM SNP.  Venn diagram showing the overlap between the transcriptomes and the proteomes in the up- and downregulated groups, respectively. The number of genes/proteins in each part is indicated. **b** Expression levels of *MAC1* and its downstream genes/proteins. **c** qRT-PCR results. Relative expressions of *MAC1* and downstream genes in SNP-treated biofilm cells compared with the control

To obtain insights into the mechanism by which NO mediates its effects in yeast biofilm cells, the transcriptome and proteome of biofilm cells treated with the NO donor SNP were analyzed. Genes expression levels of which changed by over twofold were recognized as significantly regulated. A comparison of cells that formed a biofilm under NO treatment and untreated control revealed 55 and 47 significantly up- and downregulated genes, respectively. Proteins with over 1.2-fold changes were considered significantly changed, which yielded 146 and 133 significantly up- and downregulated proteins, respectively. However, only 11 genes/proteins showed the same expression variation trend in the transcriptome and proteome (Fig. 2a). These genes/proteins were enriched for copper ion transmembrane transporter activity when classified by molecular function (*p*-value 2.875*E*−05) (Additional file 2). The *CTR1*, *CTR3,* and *FRE1* genes/proteins in the category of copper iron transport were significantly upregulated. These three genes are all regulated by transcription factor Mac1p [33], but the expression of *MAC1* was not significantly regulated in either the transcriptome or the proteome. In addition to *CTR1*, *CTR3*, and *FRE1*, other genes downstream of *MAC1—FRE7*, *IRC7,* and *REE1* showed various degrees of upregulation in the transcriptome and proteome (Fig. 2b), which was verified by qRT-PCR (Fig. 2c). This result was in agreement with a previous study showing that NO which was produced by an antioxidative mechanism activates the transcription factor Mac1p via posttranslational modification [33].

### *MAC1* influenced yeast biofilm formation by regulating the transcriptional levels of *CTR1*

**Fig. 3:**
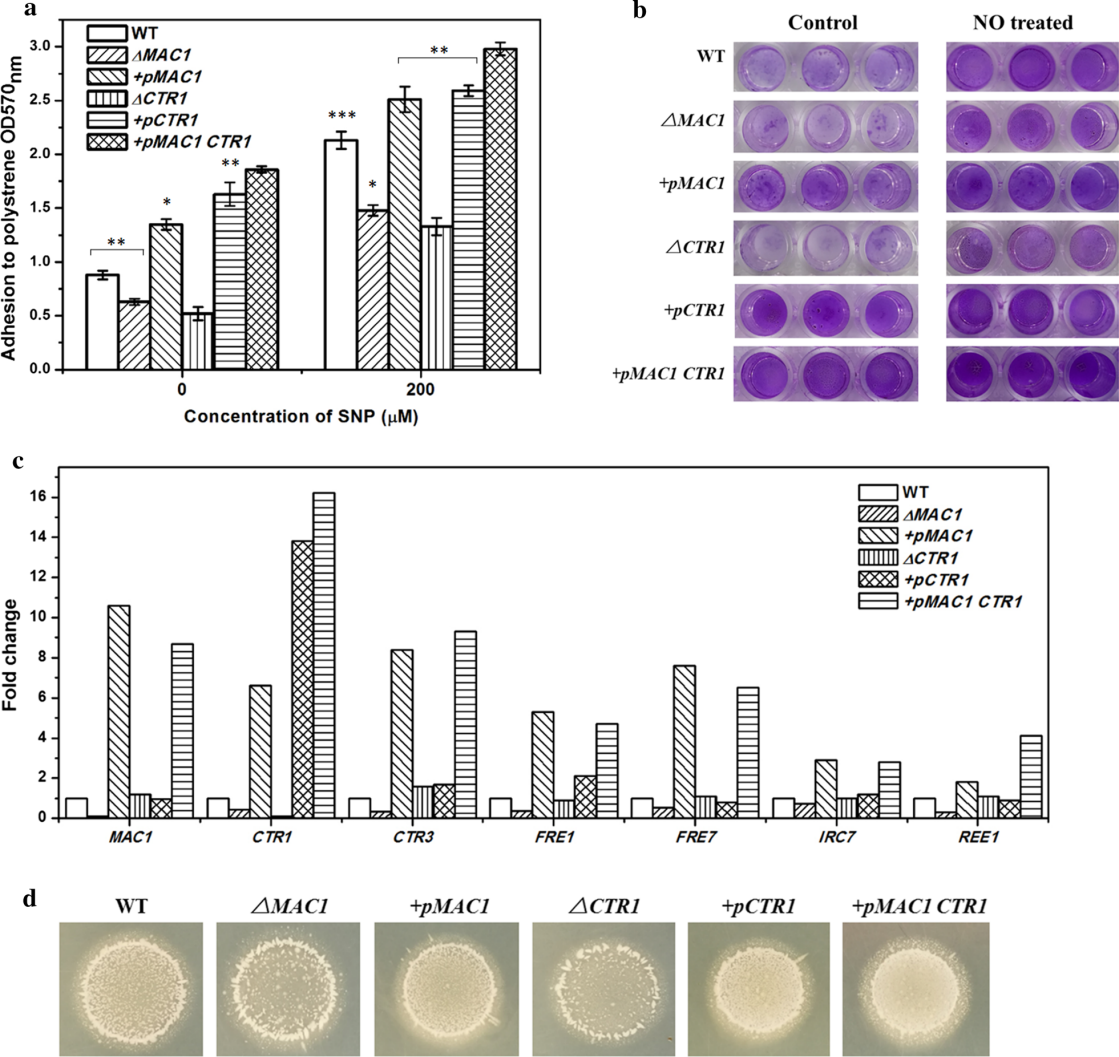
**a** Biofilms of the WT and five mutants formed in 96-well plates under control conditions and with SNP treatment. **b** Photographs of biofilms formed on plastics by WT, ∆*MAC1*, ∆*CTR1*, +*pMAC1*, +*pCTR1*, and +*p MAC1 CTR1*. **c** Relative expression of *MAC1* and downstream genes in ∆*MAC1*, ∆*CTR1*, +*pMAC1*, +*pCTR1*, and +*pMAC1 CTR1*, compared with WT. **d** Plate-wash tests of the WT, ∆*MAC1*, ∆*CTR1*, +*pMAC1*, +*pCTR1*, and +*pMAC1 CTR1*. Photos of pre- and post-washed strains were taken. The values are the means and standard deviations of three independent experiments. ***p < 0.001, **p < 0.01, *p < 0.05 by Student’s *t*-test

To explore whether *MAC1* plays a role in yeast biofilm formation under NO treatment, we deleted and overexpressed the *MAC1* gene in *S. cerevisiae* named as ∆*MAC1* and +*pMAC1*, respectively. The expression levels of *MAC1* and its downstream genes were quantified by qRT-PCR (Fig. 3c). The six genes were downregulated expressed in ∆*MAC1* in various degrees. In addition, overexpression of *MAC1* leaded upregulated expression of the six genes. The biofilm formed by these strains were quantified in 96-well plates (Fig. 3b). Compared to WT, ∆*MAC1* formed decreased and +*pMAC1* formed increased biofilm formations on plates in both the situations of control and NO treated. All strains formed stronger biofilms under NO treatment than in control (Fig. 3a). This may result from other factors, like *FLO11* which conferring biofilm formation. The expression of *FLO11* was significantly upregulated when biofilm cells treated with NO, but did not change in ∆*MAC1* or +*pMAC1* strains (Additional file 3: Figure S3). A plate-wash test was performed to evaluate the ability of invasive growth which was supposed to depend on cell-surface’s adhesive ability [30]. In this test, colonies remaining on the agar plate formed by +*pMAC1* were most, followed by WT, ∆*MAC1* finally.

Since *MAC1* influenced transcription of six genes encoding functional proteins, it seemed highly likely possible that these genes play a role in yeast biofilm formation. To test this, six deletion mutants named ∆*CTR1*, ∆*CTR3*, ∆*FRE1*, ∆*FRE7*, ∆*IRC7,* and ∆*REE1* and six overexpression mutants +*pCTR1*, +*pCTR3*, +*pFRE1*, +*pFRE7*, +*p IRC7*, and +* REE1* were constructed. Compared their biofilm formation to the WT (Fig. 3a), biofilm formed by ∆*CTR1* was pronounced reduced, while +*pCTR1* formed increased biofilm. None of other mutant strains displayed changes in biofilm formation comparing with WT (Additional file 4: Figure S4), including the strain overexpressing *CTR3* whose function is same with *CTR1* [34]. Consistent with the biofilm formation on plastics, the invasive growth of +*pCTR1* was the strongest among the six strains, followed by that of +*pMAC1* > WT > ∆*MAC1 *> ∆*CTR1* (Fig. 3d).

### The regulation of yeast biofilm formation by NO is independent of intracellular copper and iron levels

*MAC1* encodes a transcription factor which regulates the expression of genes such as *CTR1* and *CTR3* involved in copper uptake [35]. Moreover, iron levels are also controlled by *MAC1* via its regulation of the expression of *FRE1* and *FRE7*, which are responsible for the reduction of ferric iron [34]. Metal ions have been recognized as important factors that affect microbial biofilm formation [36]. To explore whether copper or iron levels mediated this regulation process, the copper and iron levels in the cells of these strains from biofilms formed under control conditions and NO treatment were determined (Table 1). Deletion of *MAC1* or *CTR1* reduced the copper and iron contents in biofilm cells. +*pMAC1* and +*pCTR1* cells absorbed more copper and iron, compared with WT. In *S. cerevisiae*, it has been found that iron transporter *Pet3* does not work until it is bound with copper [34]. Therefore, increased copper content in +*pCTR1* could improve iron content in cell. Except ∆*MAC1*, the copper and iron levels were higher in the group of NO-treated strain cells than in the controls group. This indicated that NO regulating the genes involved in copper and iron uptakes was dependent on *MAC1*. The levels of copper and iron in cells which were regulated by NO through *MAC1* may play roles in biofilm formation.

**Fig. 4:**
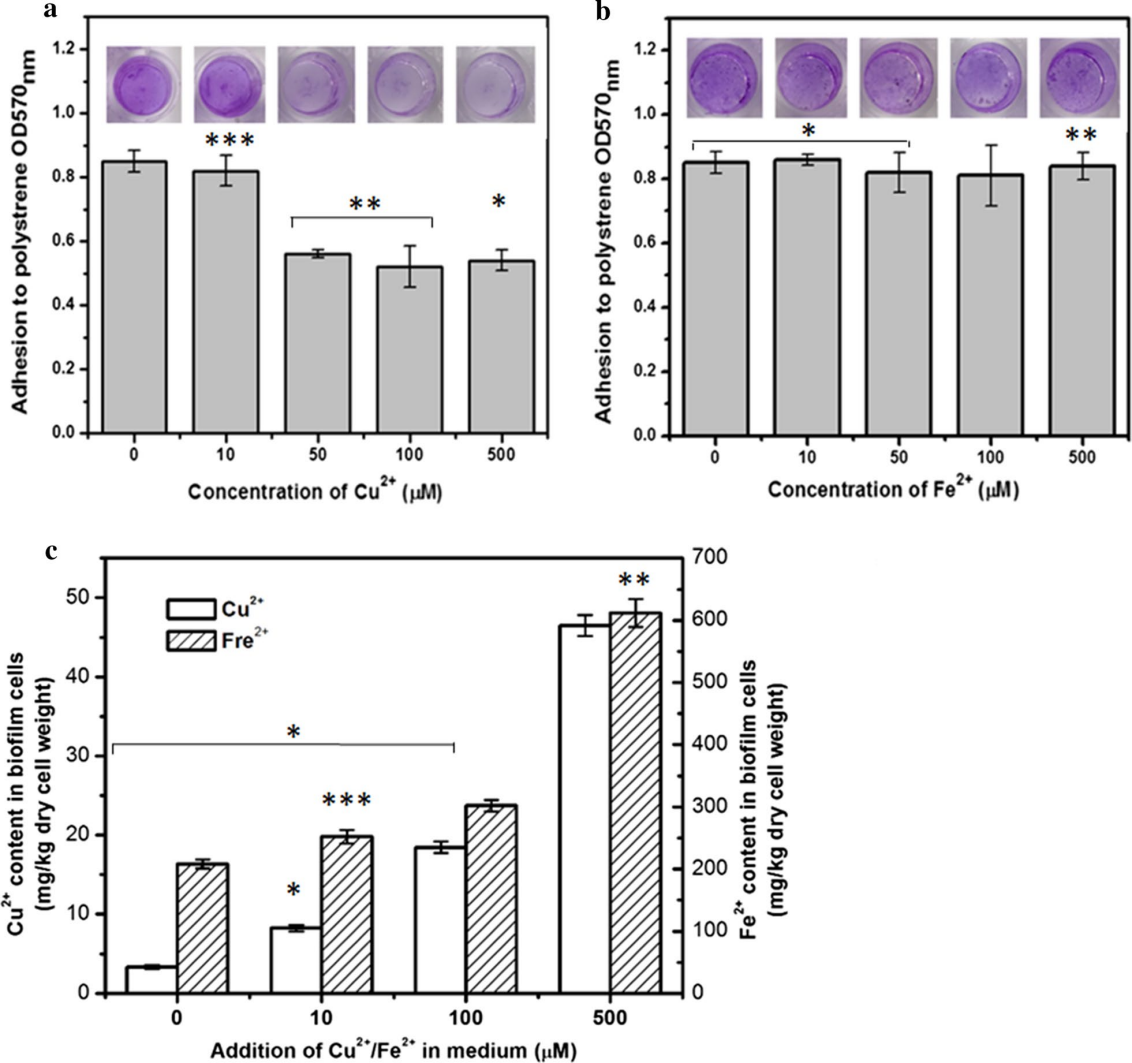
Biofilms formed in 96-well plates measured after adding different concentrations of (**a**) copper (**b**) and iron. **c** Intracellular copper and iron contents in biofilm cells were detected. The values are the means and standard deviations of three independent experiments. ***p < 0.001, **p < 0.01, *p < 0.05 by Student’s *t*-test

To confirm the role of copper and iron levels in yeast biofilm formation, we observed the biofilm formation under different concentrations of Cu^2+^ and Fe^2+^ (Fig. 4a, b). The external addition of 10 μM Cu^2+^ did not affect yeast biofilm formation. Nevertheless, when the concentration of Cu^2+^ was increased to over 50 μM, the biofilm was reduced to half of the control. Interestingly biofilms formation in the presence of added Fe^2+^ ranging from 10 to 500 μM was comparable to the control. In addition, intracellular copper and iron had been detected (Fig. 4c). With the increasing external additions of copper and iron, the copper and iron levels in biofilm cells also increased (Table 3). These findings indicated that external additions of copper and iron can be absorbed by cells.

**Table 3:** Intracellular copper and iron contents

	Strain	Control	*p*-value	Treated with 200 μM SNP	*p*-value
Copper (mg/kg dry cell weight)	WT	3.35	0.0015**	4.57	0.0007***
	*∆MAC1*	2.26	0.0064**	2.33	0.0398*
	+*pMAC1*	4.02	0.0003***	4.83	0.0229*
	*∆CTR1*	1.69	0.014*	2.04	0.0061**
	+*pCTR1*	3.87	0.0203*	4.91	0.0025**
Iron (mg/kg dry cell weight)	WT	209.42	0.0315*	650.32	0.0573
	*∆MAC1*	126.98	0.0019**	121.24	0.0028**
	+*pMAC1*	517.40	0.0382*	804.91	0.0367*
	*∆CTR1*	171.86	0.0006***	435.01	0.0448*
	+*pCTR1*	236.59	0.0197*	636.48	0.0732

The copper and iron contents in WT and mutant strains biofilm cells were detected after 6 h of culture in the absence or the presence of SNP. The data are shown as milligrams of copper and iron per kg of cell dry weight. Each value is an average of three replicates

*** *p* < 0.001, ** *p *< 0.01, * *p *< 0.05 by Student’s *t*-test

### The functions of *MAC1* and *CTR1* in ethanol resistance and fed-batch fermentation

**Fig. 5:**
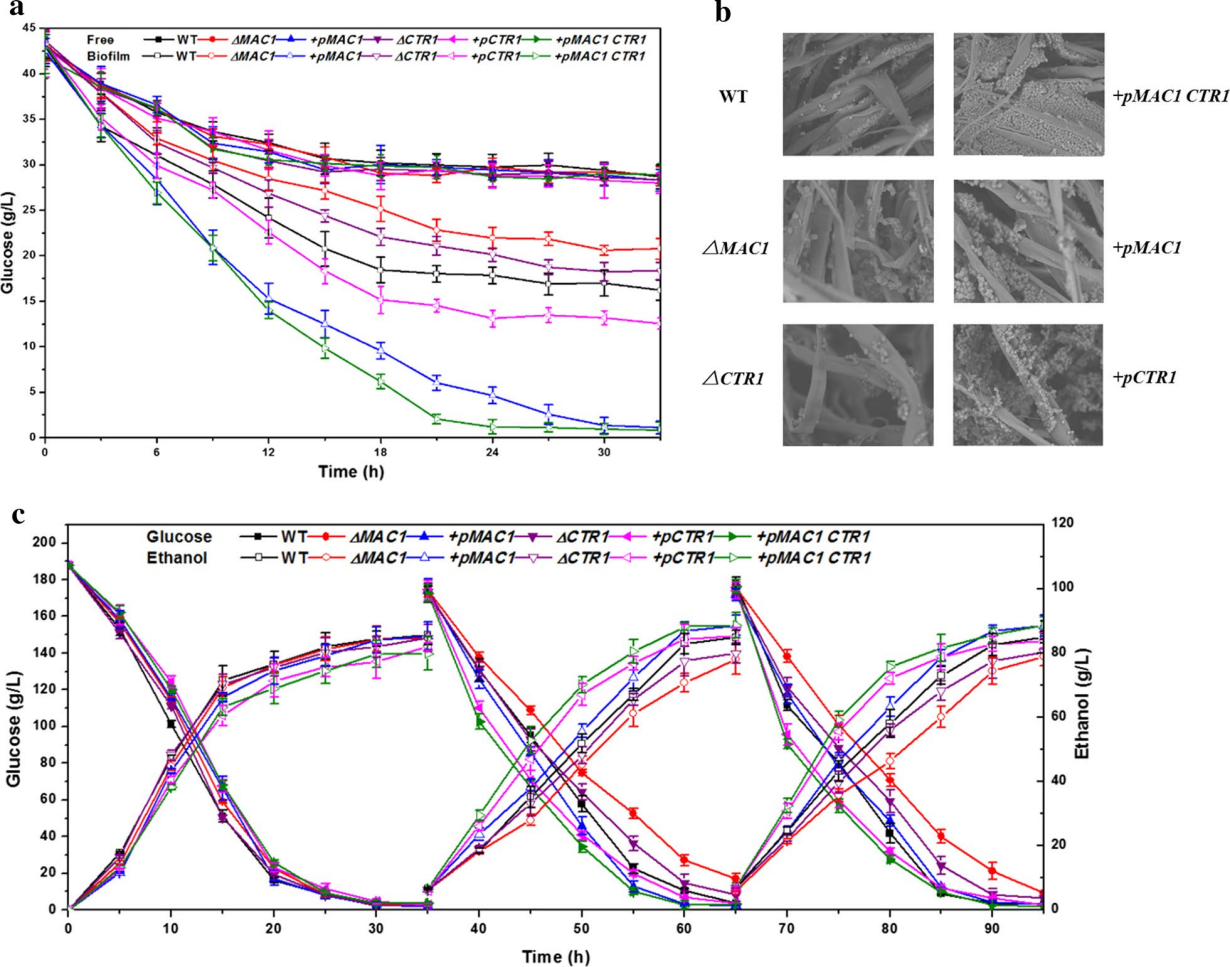
**a** Change of glucose concentration during fermentation in 10% (v/v) ethanol in free and biofilm fermentation. **b** SEM images of biofilms formed on cotton fibers by WT, ∆*MAC1*, ∆*CTR1*, +*pMAC1*, +*pCTR1, and* +*pMAC1 CTR1* after 33 h of fermentation in 10% ethanol. **c** Kinetics of batch fermentation of the three strains in immobilized cultures

To explore the effect of enhanced biofilm formation by *MAC1* and *CTR1* on ethanol resistance, we investigated the abilities of the corresponding mutants to survive during the accumulation of high amounts of ethanol by measuring the residual glucose concentrations. Glucose was depleted within 20 and 12 h in free- and biofilm fermentation without ethanol by all the strains, and there was no obvious difference among these strains in free or biofilm fermentation (Additional file 5: Figure S5). By contrast, in the presence of 10% (v/v) ethanol, glucose consumption was slower than without ethanol, and about 28 g/L of glucose remained in all the samples at 33 h in free fermentation. The glucose consumption rates of these strains in biofilm fermentation were faster than in free fermentation (Fig. 5a). In biofilm fermentation, +*pMAC1* exhausted the glucose within 33 h, while 17, 19, 21, and 13 g/L of glucose remained in the WT, ∆*CTR1*, *∆MAC1*, and +*pCTR1* samples, respectively. As *MAC1* and *CTR1* were beneficial for cell ethanol resistance, we co-expressed *MAC1* and *CTR1* in WT named as +*pMAC1 CTR1*. Besides forming the most biofilm and showing the strongest invasive growth among all the strains (Fig. 3a, b, d), +*pMAC1 CTR1* consumed glucose in the fastest way in the presence of ethanol (Fig. 5c).

Differences of glucose consumption were also observed in our immobilized fed-batch fermentation (Fig. 5c). In the first batch, +*pCTR1* and +*pMAC1 CTR1* consumed glucose and produced ethanol slightly slower than the wild-type, and other strains performed nearly same fermentation. In the next two batches, ∆*CTR1* and ∆*MAC1* consumed glucose and produced ethanol slower than WT, especially ∆*MAC1*. The fermentation process of +*pCTR1* and +*pMAC1 CTR1* were expedited and became the fastest among these strains. Compare with the WT, the speed of fermentation of +*pMAC1* was little higher. It was interesting that the +*pMAC1* and +*pMAC1 CTR1* produced about 4 g/L ethanol more than the WT. After the first batch, the biofilms formed by the three strains on cotton fibers were imaged by SEM (Fig. 5b). It could be observed that +*pMAC1 CTR1* formed the largest amounts of biofilm, followed by +*pCTR1 *> + *pMAC1 *> WT> ∆*MAC1 *> ∆*CTR1.*

## Discussion

Nitric oxide acts as signaling molecule that regulates biofilm formation in various organisms, including important pathogens [1, 4]. Due to the observation of NO triggering biofilm dispersal, it was usually studied in conjunction with antibiotics as an attempt to remove bacterial infection [26]. However, biofilm formation is enhanced by NO in some microbes, which could be beneficial in industrial biofilm reactors. In our studies, low concentrations of NO were found to contribute to biofilm formation in *S. cerevisiae*. Furthermore, increased biofilm formation in *S. cerevisiae* improves ethanol resistance of cells, which is beneficial in ethanol fermentation [30]. However, the regulation mechanism by which NO influences yeast biofilm formation had not been explored.

In this study, we studied the effect of NO on biofilm formation in *S. cerevisiae* by adding NO donors SNP and NOC-18. Low concentrations of NO enhanced *S. cerevisiae* biofilm formation both on cotton fibers and on plastics. Yeast biofilm formation was enhanced with the increasing concentration of SNP and NOC-18 from 0 to 300 μM. At the same concentration of SNP and NOC-18, the biofilm formed under SNP stimulation was stronger than the one obtained with NOC-18. This is most likely caused by differences of the NO release rate between the two donors, since the half-life of SNP (about 10–30 min) is much shorter than that of NOC-18 (about 20–56 h) [37]. In addition, the NO scavenger PTIO relieved the effect of SNP on yeast biofilm formation, while low concentrations of NaNO_2_ and NaNO_3_ did not influence biofilm formation of *S. cerevisiae*, indicating that NO itself is responsible for the effect, as opposed to other degradation products of the donors or NO derivatives.

A combined analysis of the transcriptome and proteome revealed that genes/proteins downstream of the transcription factor of Mac1p were upregulated to varying degrees. However, the RNA levels of *MAC1* did not indicate significant differential expression. This result was consistent with previous studies, which found that NO was produced in antioxidative mechanisms to activate Mac1p without affecting its expression [33]. Our results indicated that the overexpression of *MAC1* increased yeast biofilm formation and invasive growth. This was in agreement with previous studies which showed ectopic expression of *CaMAC1*, the *MAC1* homolog from *C. albicans*, promoted invasive growth in *S. cerevisiae* [38]. The ability of invasive growth depends on cell–substrate adhesion, which plays a vital role in biofilm formation [30].

Mac1p is a copper-sensing transcription factor, which is activated in response to low copper levels and is responsible for copper uptake [39]. In addition, copper has been reported to influence biofilm formation in *Legionella pneumophila* and *Pseudomonas pseudoalcaligenes* [36, 40]. Consistent with the antioxidative mechanism in which cellular copper levels were increased under NO treatment [35], the cellular copper levels increased in yeast biofilm cells under NO treatment. This may suggest that high copper levels contribute to biofilm formation regulated by NO. However, the addition of low concentrations of Cu^2+^ in the medium did not promote yeast biofilm formation, suggesting that the increase of copper levels regulated by *MAC1* under NO stimulation is not directly related to biofilm formation. However, since the activity of Mac1p can be activated by copper depletion, it could be ruled out that its activity was affected by cellular copper levels in the biofilm formation process. Other posttranslational modifications that are known to activate Mac1p, such as S-nitrosylation and phosphorylation [33], may be involved in this regulation process. The addition of more than 50 μM copper reduced biofilm formation, which may have resulted from the inactivation of Mac1p in response to high copper levels. Although iron levels were higher in the NO-treated biofilm cells than in the controls, exogenous micromolar iron did not influence yeast biofilm formation, suggesting that high iron levels were not responsible for increased biofilm formation under NO treatment. Because SNP contains Fe(II), and adding iron reduced yeast biofilm formation, the NO-regulated yeast biofilm formation could not have resulted from exogenous iron stemming from SNP.

*FLO11*, encoding a flocculation protein conferring cell–substrate adhesion, plays a key role in biofilm formation [30]. However, in ∆*MAC1* or +*pMAC1* strains, the flocculation gene *FLO11* was not differentially expressed compared with the WT (Additional file 3: Figure S3), indicating that *MAC1* regulated yeast biofilm formation without influencing *FLO11* expression. The six genes downstream of *MAC1*, especially *CTR1* and *CTR3*, were upregulated in +*pMAC1* as well as in biofilm cells treated with NO. Among the six genes transcriptionally activated by Mac1p, only *CTR1* influenced yeast biofilm formation. *CTR1* and *CTR3* encode two transmembrane proteins that are responsible for high-affinity copper transport [39]. The expression level of *CTR1* affected yeast biofilm formation, while *CTR3* did not. Conversely, a *CTR1* deletion mutant of *S. cerevisiae* was found to have decreased biofilm formation in a genome-wide screening [41]. Although the two proteins are functionally the same and share little homology in amino acid sequence, they are structurally distinct [35]. The unique function of Ctr1p may result from its special structure. In the N-terminal extracellular domain, Ctr1 protein is highly glycosylated and has eight repeats of the Mets domains (MXXXM motif, X representing a hydrophobic residue) [42]. Overexpressed Ctr1p could improve the hydrophobicity of yeast cells. It was reported that Cell surface hydrophobicity has been proved to be a good predictor of biofilm formation [43]. The conserved hydrophobicity of this domain may contribute to yeast biofilm formation. In addition, yeast two-hybrid experiments demonstrated that the N-terminal extracellular domain of Ctr1p supports self-interaction that is not modulated by copper [44]. We speculated that the N-terminal extracellular domains of Ctr1p from different cells could interact with each other to increase cell–cell adhesion. Ctr1p may function as Flo11p which is a cell wall protein and required in biofilm formation [30]. In addition, +*pCTR1* showed stronger biofilm formation than +*pMAC1*, and the transcription level of *CTR1* was higher in +*pCTR1* biofilm cells than in those of +*pMAC1*. ∆*CTR1*, in which no *CTR1* was expressed, showed weaker ability of biofilm formation than ∆*MAC1* in which *CTR1* expressed at a relatively lower level. It was likely the biofilm formation was dependent on the expression of *CTR1*. The phenotype and transcription were consistent and illustrated that *CTR1* plays a significant role in yeast biofilm formation.

In the presence of 10% ethanol, the six strains all consumed glucose slowly in free fermentation, illustrating that the expression of *MAC1* and *CTR1* did not direct effect ethanol resistance of cells. +*pMAC1 CTR1* which had the strongest ability of biofilm consumed glucose fastest among these strains, and all stains all consumed glucose faster in biofilm form than in free fermentation, indicating that biofilm formation plays the key role in ethanol resistance. It was interesting that +pMAC1 displayed greater glucose consumption than +*pCTR1* in this experiment, which was opposite to the biofilm formation of the two mutant strains. In addition to the two *CTR* genes, *FRE1* and *FRE7*, encoding ferric reductase transmembrane components involved iron uptake [34], were upregulated in +*pMAC1*. The difference of ethanol resistance between the two mutants may have resulted from the higher iron content in +*pMAC1* than in +*pCTR1* (Table 1), since iron was found to upregulate superoxide dismutase (SOD) in proteome analysis [45], and SOD can protect cells from oxidative stress resulting from ethanol toxicity [46]. As a consequence, +*pMAC1* had superior ethanol resistance than +*pCTR1* although +*pCTR1* displayed stronger biofilm formation. This was also why ∆MAC1 showed weaker ethanol resistance than ∆CTR1. In immobilized fed-batch fermentation, increased biofilm formation of overexpressed mutant strains led to quicker glucose consumption and shortened the fermentation cycle in the later batches. This was especially true for +*pMAC1 CTR1*, which displayed the strongest biofilm formation and ethanol resistance among these strains. The higher activity of ∆*CTR1* cells than those of ∆*MAC1* may be explained by the downregulation of SOD in ∆*MAC1*.

## Conclusion

The study focused on exploring the effects of the signal molecule NO on yeast biofilm formation during immobilized fermentation. Our results indicate that NO indeed contributes to biofilm formation in *S. cerevisiae*. A mechanistic investigation of this relationship revealed that the transmembrane protein Ctr1p, which is transcriptionally activated by Mac1p, contributed to yeast biofilm formation. The findings presented in this paper contribute to the understanding of the mechanisms of NO-mediated regulation in *S. cerevisiae* biofilm formation. The important pathways and factors identified in this study can be applied to regulate biofilm formation in immobilized fermentation. However, more work is necessary to explore the mechanism of increased activity of Mac1p following NO treatment and any other regulatory pathways through which NO may enhance yeast biofilm formation.

## Additional files

**Additional file 1: Figure S1.** Kinetics of biofilm fermentation in the presence of different concentrations of SNP.

**Additional file 2.** Gene ontology enrichment analysis of genes/proteins which showed the same expression variation trend in the transcriptome and proteome comparison for biofilm cells under control and NO treated.

**Additional file 3: Figure S3.** The expression of *FLO11* in mutant strains in control and NO treatment compared with WT.

**Additional file 4: Figure S4.** The biofilm formed by ∆*CTR3*, ∆*FRE1*, ∆*FRE7*, ∆*IRC7*, ∆*REE1*, +*pCTR3*, +*pFRE1*, +*pFRE7*, +*pIRC7 and* +*pREE1* in control and NO treatment. The values are the means and standard deviations of three independent experiments. ***p < 0.001, **p < 0.01, *p < 0.05 by Student’s *t*-test.

**Additional file 5: Figure S5.** Change of glucose concentration during fermentation of the three strains in free and biofilm states.

## Abbreviations

NO: nitric oxide
QS: quorum sensing
c-di-GMP: cyclic di-GMP
cGMP: cyclic GMP
SNP: sodium nitroprusside
NOC-18: diethylenetriamine NONOate
cPTIO: carboxy-PTIO
SEM: scanning electron microscopy
SOD: superoxide dismutase
