# Supplementary figures and images for: Nitric oxide increases biofilm formation in Saccharomyces cerevisiae by activating the transcriptional factor Mac1p and thereby regulating the transmembrane protein Ctr1
Source: Biotechnol Biofuels. 2019 Feb 14;12:30. doi: 10.1186/s13068-019-1359-1 (PMC6375214; doi:10.1186/s13068-019-1359-1)

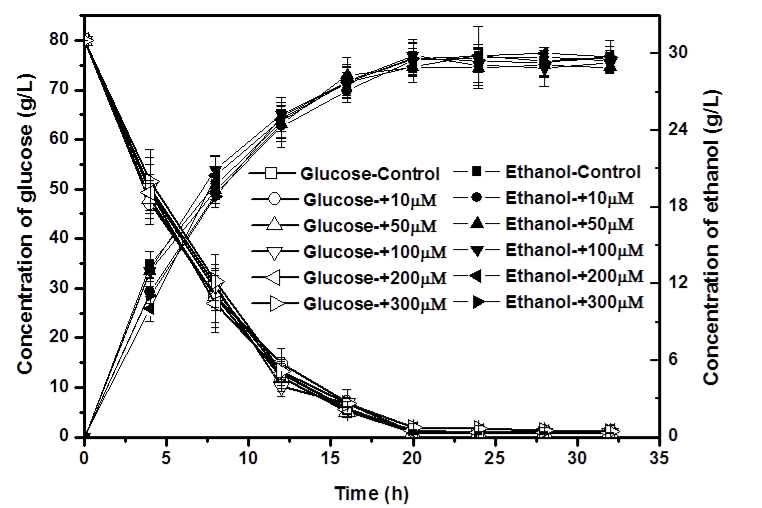

Supplement: Supplementary file 1 — Additional file 1: Figure S1. Kinetics of biofilm fermentation in the presence of different concentrations of SNP. [file 13068_2019_1359_MOESM1_ESM.tif]

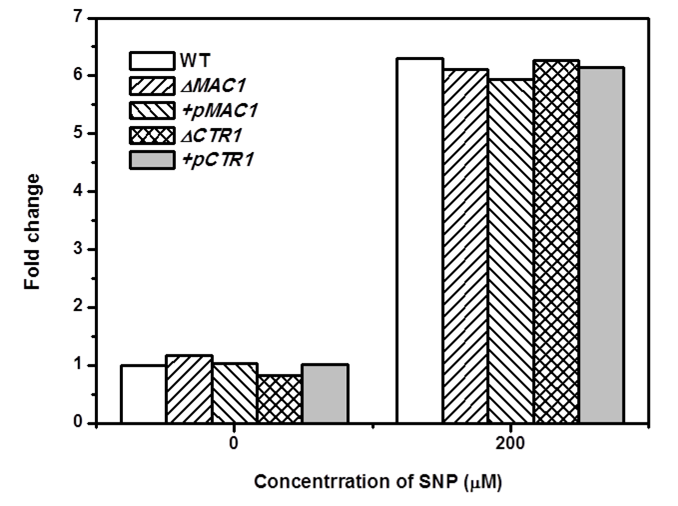

Supplement: Supplementary file 3 — Additional file 3: Figure S3. The expression of FLO11 in mutant strains in control and NO treatment compared with WT. [file 13068_2019_1359_MOESM3_ESM.tif]

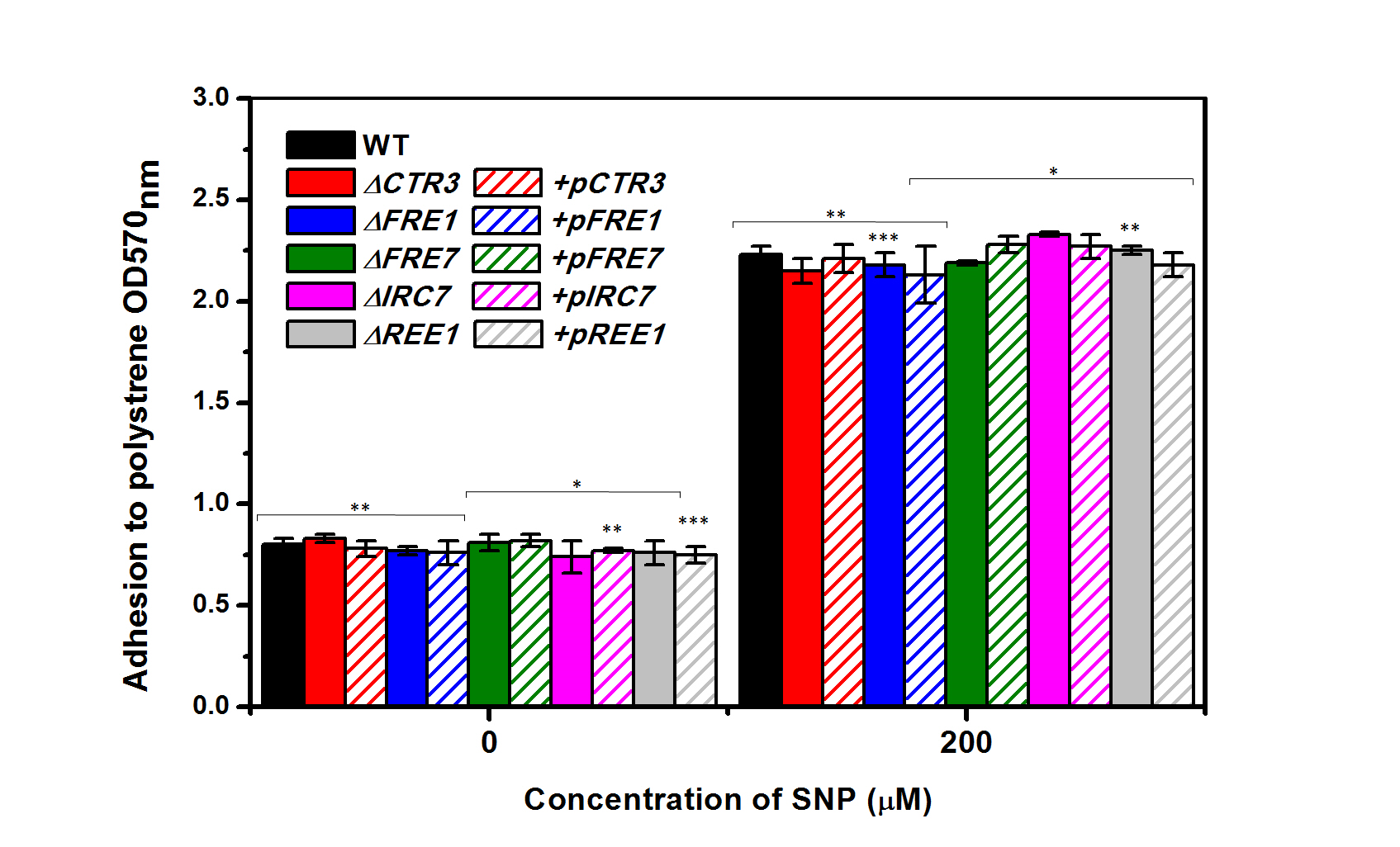

Supplement: Supplementary file 4 — Additional file 4: Figure S4. The biofilm formed by ∆CTR3, ∆FRE1, ∆FRE7, ∆IRC7, ∆REE1, +pCTR3, +pFRE1, +pFRE7, +pIRC7 and +pREE1 in control and NO treatment. The values are the means and standard deviations of three independent experiments. ***p < 0.001, **p < 0.01, *p < 0.05 by Student’s t-test. [file 13068_2019_1359_MOESM4_ESM.tif]

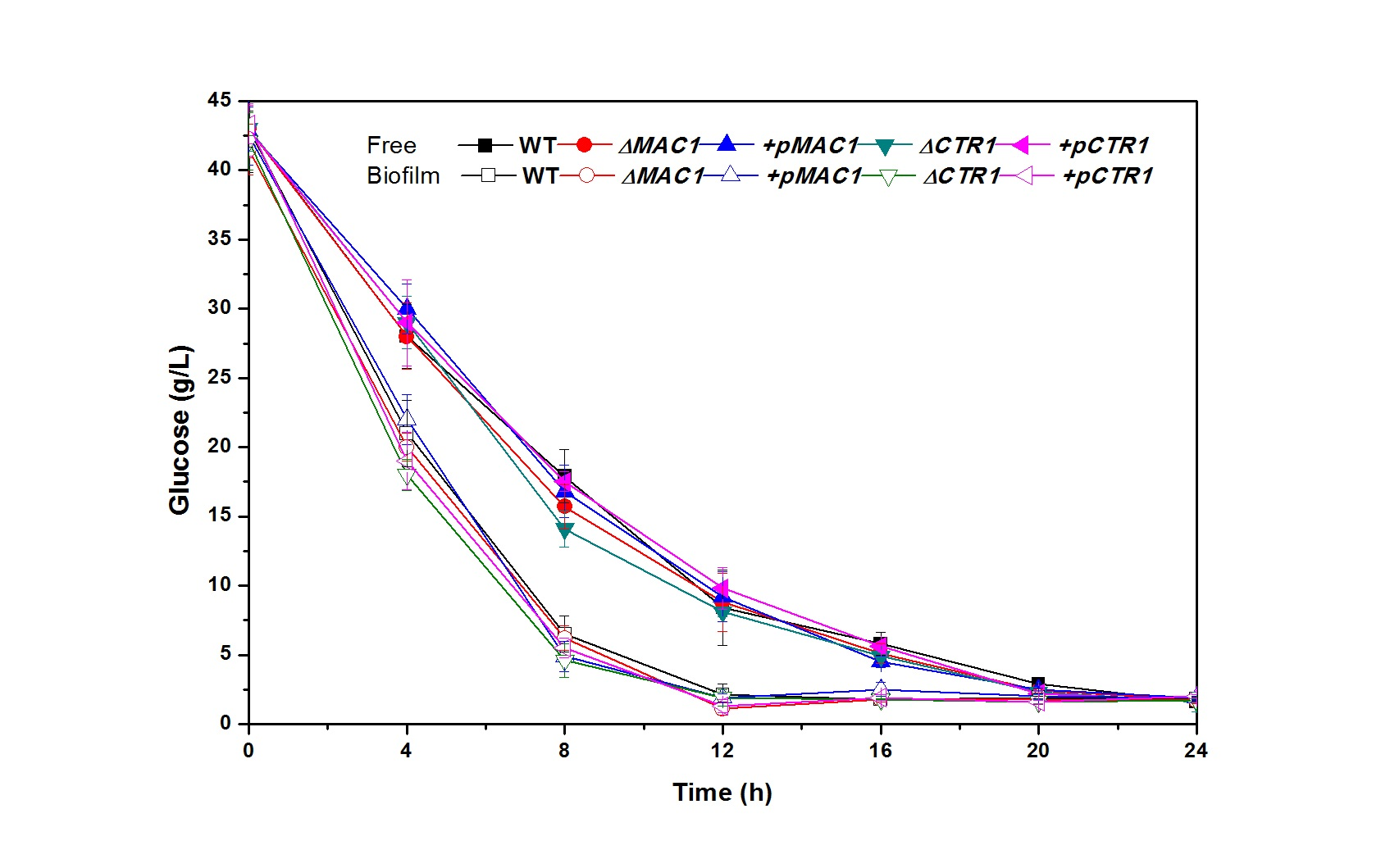

Supplement: Supplementary file 5 — Additional file 5: Figure S5. Change of glucose concentration during fermentation of the three strains in free and biofilm states. [file 13068_2019_1359_MOESM5_ESM.tif]
